# Functional characterization of aconitase X as a *cis*-3-hydroxy-L-proline dehydratase

**DOI:** 10.1038/srep38720

**Published:** 2016-12-08

**Authors:** Seiya Watanabe, Kunihiko Tajima, Satoshi Fujii, Fumiyasu Fukumori, Ryotaro Hara, Rio Fukuda, Mao Miyazaki, Kuniki Kino, Yasuo Watanabe

**Affiliations:** 1 Department of Bioscience, Graduate School of Agriculture, Ehime University, 3-5-7 Tarumi, Matsuyama, Ehime 790-8566, Japan; 2 Faculty of Agriculture, Ehime University, 3-5-7 Tarumi, Matsuyama, Ehime 790-8566, Japan; 3 Center for Marine Environmental Studies (CMES), Ehime University, 2-5 Bunkyo-cho, Matsuyama, Ehime 790-8577, Japan; 4 Department of Bio-molecular Engineering, Graduate School of Science and Technology, Kyoto Institute of Technology, Matsugasaki, Sakyo-ku, Kyoto 606-8585, Japan; 5 Faculty of Frontiers of Innovative Research in Science and Technology (FIRST), Konan University, 7-1-20 Minatojima-minamimachi, Chuo-ku, Kobe, Hyogo 650-0047, Japan; 6 Faculty of Food and Nutritional Sciences, Toyo University, 1-1-1 Izumino, Itakura-machi, Ora-gun, Gunma 374-0193, Japan; 7 Research Institute for Science and Engineering, Waseda University, 3-4-1 Ohkubo, Shinjuku-ku, Tokyo 169-8555, Japan; 8 Department of Applied Chemistry, Faculty of Science and Engineering, Waseda University, 3-4-1 Ohkubo, Shinjuku-ku, Tokyo 169-8555, Japan

## Abstract

In the aconitase superfamily, which includes the archetypical aconitase, homoaconitase, and isopropylmalate isomerase, only aconitase X is not functionally annotated. The corresponding gene (*LhpI*) was often located within the bacterial gene cluster involved in L-hydroxyproline metabolism. Screening of a library of (hydroxy)proline analogues revealed that this protein catalyzes the dehydration of *cis*-3-hydroxy-L-proline to Δ^1^-pyrroline-2-carboxylate. Furthermore, electron paramagnetic resonance and site-directed mutagenic analyses suggests the presence of a mononuclear Fe(III) center, which may be coordinated with one glutamate and two cysteine residues. These properties were significantly different from those of other aconitase members, which catalyze the isomerization of α- to β-hydroxy acids, and have a [4Fe-4S] cluster-binding site composed of three cysteine residues. Bacteria with the *LhpI* gene could degrade *cis*-3-hydroxy-L-proline as the sole carbon source, and *LhpI* transcription was up-regulated not only by *cis*-3-hydroxy-L-proline, but also by several isomeric 3- and 4-hydroxyprolines.

L-Hydroxyproline has been found in certain proteins, particularly in collagen, and in some peptide antibiotics. In mammalian systems, the L-proline residue is post-translationally hydroxylated to *trans*-4-hydroxy-L-proline [(2*S*,4*R*)-4-hydroxyproline] or *trans*-3-hydroxy-L-proline [(2*S*,3*S*)-3-hydroxyproline] by a diverse set of prolyl 4-hydroxylase (EC 1.14.11.2) and prolyl 3-hydroxylase (EC 1.14.11.7) with different protein substrates, respectively^1^. A number of bacterial and fungal enzymes are known to directly hydroxylate free L-proline to *trans*-4-hydroxy-L-proline^2^, *trans*-3-hydroxy-L-proline^3^,^4^, *cis*-4-hydroxy-L-proline [(2*S*,4*S*)-4-hydroxyproline]^5^, or *cis*-3-hydroxy-L-proline [(2*S*,3*R*)-3-hydroxyproline]^6^, which are also found as components of several peptide antibiotics produced by these microorganisms^7^,^8^,^9^,^10^,^11^.

Among the several stereoisomers of L-hydroxyproline, *trans*-4-hydroxy-L-proline is the most common in nature, and is converted to pyruvate and glyoxylate via three intermediates in mammals (Table S1)^1^. On the other hand, a number of bacteria, including *Pseudomonas aeruginosa* PAO1 and *Pseudomonas putida* KT2440, metabolize *trans*-4-hydroxy-L-proline to α-ketoglutarate by four enzymes (encoded by *LhpA*~*F* genes) (Fig. 1a and Table S1)^12^,^13^. In contrast to *trans*-4-hydroxy-L-proline, the metabolic pathways of *trans*-3-hydroxy-L-proline in bacteria and mammalians may be similar, and consist of a dehydratase (*LhpG*) and a reductase (*LhpH* or *LhpK*), by which *trans*-3-hydroxy-L-proline is converted to L-proline via Δ^1^-pyrroline-2-carboxylate (Fig. 1b and Table S1)^14^,^15^. It is likely that the reductase is also involved in *cis*-3-hydroxy-L-proline metabolism in bacteria, in which Δ^1^-pyrroline-2-carboxylate is produced by a different dehydratase than the LhpH protein (*LhpJ*) (Fig. 1c and Table S1)^16^. These *LhpA*~*K* genes often cluster together with the putative L-hydroxyproline (ABC-type) transporter genes, transcriptional regulator gene, aconitase-like gene (*LhpI*) and proline racemase-like gene (*LhpL*) on bacterial genomes (Fig. 1d; referred to as the “L-hydroxyproline gene cluster”)^12^,^13^,^15^,^16^. Among these hypothetical genes, it is likely that the *LhpI* gene (protein) in particular is not necessary for the known pathway of L-hydroxyproline metabolism due to its putative function, based on amino acid sequence similarity.

The aconitase superfamily contains four functional hydro-lyase enzymes: aconitase (EC 4.2.1.3; Acn), 2-methylcitrate dehydratase (EC 4.2.1.79; AcnD), homoaconitase (EC 4.2.1.114; HACN), and isopropylmalate isomerase (EC 4.2.1.33; IPMI), which have been classified into eight phylogenetic subfamilies: (1) AcnA of bacteria^17^; (2) AcnB of bacteria^18^; (3) mitochondrial Acn (mAcn)^19^; (4) cytoplasmic Acn and iron regulatory protein (IRP) of mammalians^20^; (5) AcnD of bacteria^21^; (6) HACN of bacteria and archaea^22^,^23^; (7) IPMI of bacteria, archaea, and fungi^24^,^25^,^26^; (8) function unknown aconitase X (AcnX) (Fig. 2). All of these subfamilies (referred to as “Acn enzymes”), except for the last one, catalyze the homologous stereospecific isomerization of α- to β-hydroxyl acids by sequential dehydration and hydration (*anti*-elimination/addition), and their active centers contain a [4Fe-4S] iron-sulfur cluster. By contrast, AcnX (subfamily) was initially discovered by a comparative analysis of archaeal genomes in 2003^27^. Although the secondary structural elements of Acn enzymes may also be conserved in this subfamily, the sequence similarities between them are insignificant. The AcnX subfamily has been further classified into “AcnX_Type I_”, consisting of a single polypeptide from bacteria, and “AcnX_Type II_” found in a number of bacteria (AcnX_Type IIa_) and archaea (AcnX_Type IIb_), which consists of (fragmented) small and large polypeptide chains. The small polypeptide (subunit) of AcnX_Type II_ is homologous to the so-called “fourth domain” of Acn enzymes as well as the IPMI and HACN of bacteria and archaea (Fig. 2)^22^,^23^,^24^,^25^. Among the three AcnX subfamilies, LhpI protein corresponds to AcnX_Type I_.

We report here that LhpI protein catalyzes the dehydration of *cis*-3-hydroxy-L-proline to Δ^1^-pyrroline-2-carboxylate, by a reaction that was not homologous with those of known Acn enzymes, and contains mononuclear Fe(III), but not the [4Fe-4S] cluster. Bacterial growth and transcriptional analysis suggested that this enzyme is surely involved in *cis*-3-hydroxy-L-proline metabolism.

## Results and Discussion

### Identification of substrates and functions

**Figure 1:**
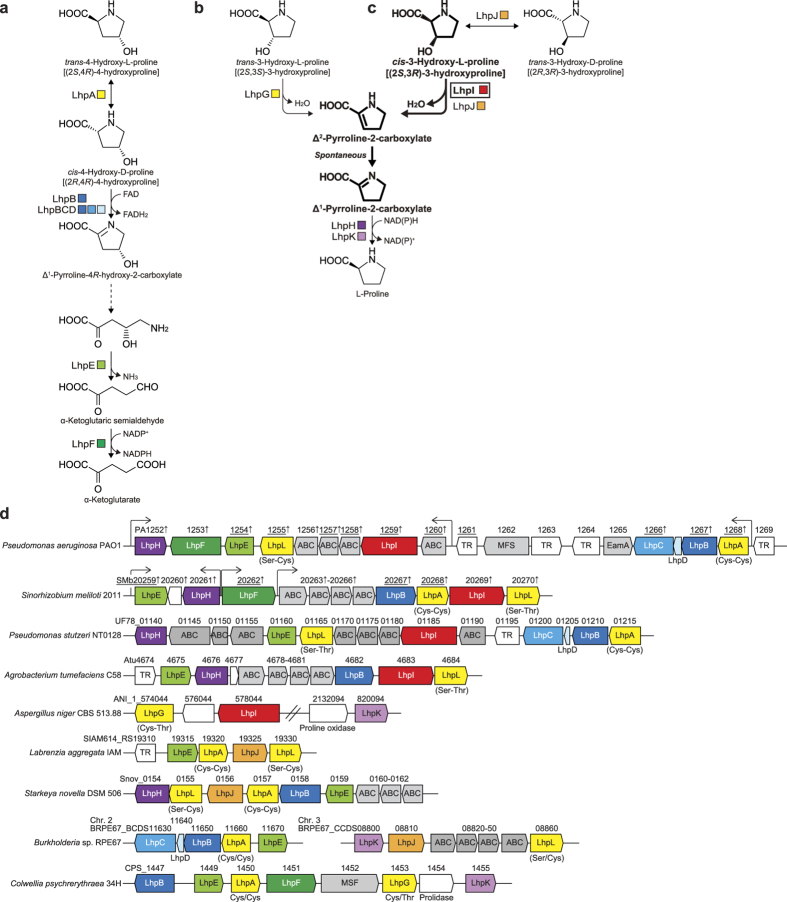
L-Hydroxyproline metabolism in bacteria. (**a**) *trans*-4-Hydroxy-L-proline, (**b**) *trans*-3-hydroxy-L-proline, and (**c**) *cis*-3-hydroxy-L-proline pathways. (**d**) Schematic gene clusters related to the metabolism of L-hydroxyproline. Enzyme names and EC numbers are listed in Table S1. Homologous genes are indicated in the same color and correspond to Fig. 1(**a**,**b,c**). A characteristic pair of catalytic amino acid residues of proline racemase superfamily enzymes are shown in the bottom of the *LhpA, LhpG*, and *LhpL* genes (see “Supplementary Discussion”). Gray and TR genes are putative L-hydroxyproline transporters and transcriptional regulators, respectively. Genes with up arrows from *P. aeruginosa* PAO1 and *S. meliloti* 2011 are induced by *trans*-4-hydroxy-L-proline^28^,^29^. Mutant strains of the underlined genes cannot grow on *trans*-4-hydroxy-L-proline as a sole carbon source.

A genetic analysis of the L-hydroxyproline gene cluster (PA1252-1269) from *Pseudomonas aeruginosa* PAO1 was recently reported in detail (Fig. 1d)^28^. Among the 18 components, 10 genes including PA1259 (*LhpI*) were significantly induced by *trans*-4-hydroxy-L-proline. Furthermore, the *LhpI* mutant, constructed by transposon insertion, as well as mutants of the known *trans*-4-hydroxy-L-proline metabolic gene lost the ability to grow on *trans*-4-hydroxy-L-proline. However, this phenotype may be due to a polar effect on downstream genes^28^. The non-polar *LhpI* deletion mutant (SMb20269; Fig. 1d) of *Sinorhizobium meliloti* 1021 was previously reported to grow normally on *trans*-4-hydroxy-L-proline^29^. Therefore, there has been no evidence to show that the *LhpI* gene plays a role in the metabolism of L-hydroxyproline.

**Figure 3:**
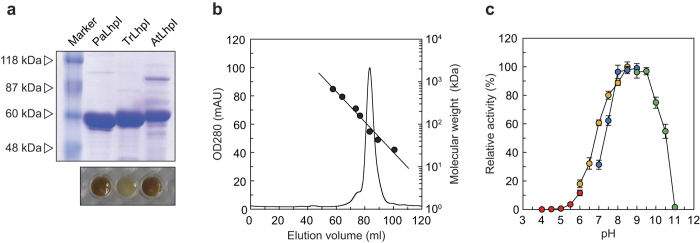
Properties of purified LhpI proteins. (**a**) SDS-PAGE analysis of recombinant proteins (5 μg in a 10% (w/v) gel). The theoretical molecular masses of PaLhpI, AtLhpI, and TrLhpI are 63354.77, 60389.43, and 65215.77, respectively. The bottom panel is a photograph of LhpI proteins (~30 mg/ml). (**b**) Elution profile of PaLhpI during gel filtration. The molecular mass, 67 kDa, was determined by using the following equation: *y* = 80855e^−0.082*x*^ (*R*^2^ = 0.9784). (**c**) Effects of pH on the activity of PaLhpI. Reactions were buffered with 50 mM acetic acid-NaOH (pH 4.0–6.0) (red), 50 mM potassium phosphate (pH 6.0–8.5) (orange), 50 mM Tris-HCl (pH 7.0–9.0) (blue), and 50 mM glycine-NaOH (pH 9.0–11.0) (green), instead of 50 mM Tris-HCl (pH 8.0) used under standard assay conditions. Values are the average ± SD, *n* = 3.

**Figure 4:**
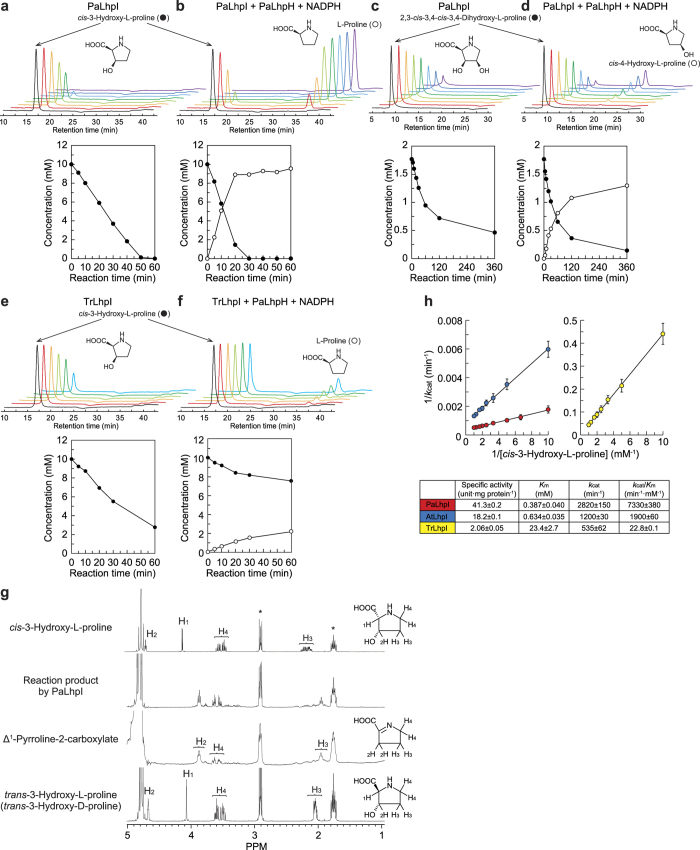
Characterization as a *cis*-3-hydroxy-L-proline dehydratase. Analysis of reaction products of *cis*-3-hydroxy-L-proline (**a**,**b,e,f**) and 2,3-*cis*-3,4-*cis*-dihydroxy-L-proline (**c**,**d**) by PaLhpI (**a**,**c**), PaLhpI + PaLhpH + NADPH (**b**,**d**), TrLhpI (**e**), or TrLhpI + PaLhpH + NADPH (**f**) using an amino acid analyzer. Concentrations are expressed as the percentage of the amount initially present. Three independent experiments were performed, and the typical result is shown. (**g**) ^1^H NMR spectra of *cis*-3-hydroxy-L-proline, the reaction product of *cis*-3-hydroxy-L-proline by PaLhpI, Δ^1^-pyrroline-2-carboxylate, and *trans*-3-hydroxy-L-proline (identical to *trans*-3-hydroxy-D-proline). Asterisks are peaks derived from an internal standard. Left panels show the assignments of protons in D_2_O. (**h**) Lineweaver-Burk plots and kinetic parameters of PaLhpI (red), AtLhpI (blue), and TrLhpI (yellow) toward *cis*-3-hydroxy-L-proline. Values are the average ± SD, *n* = 3.

The recombinant (His)_6_-tagged PaLhpI protein was successfully expressed in *P. putida* cells, and purified to homogeneity using a nickel-chelating affinity column (Fig. 3a,b). The apparent molecular masses, estimated by SDS-PAGE and analytic gel filtration, were 60 and 67 kDa, indicating a monomeric structure. Initially, no hydro-lyase activity toward citrate and/or *cis*-acotinate was observed in this protein. Therefore, in the next approach for functional analysis, eight proline derivatives (10 mM; Fig. S1) were tested as substrates in Tris-HCl buffer (pH 8.0) without additives. Among them, only *cis*-3-hydroxy-L-proline was consumed in a time-dependent manner (specific activity of 39.5 μmol·min^−1^·mg^−1^) (Fig. 4a), and the reaction product was identified as Δ^1^-pyrroline-2-carboxylate by ^1^H NMR (Fig. 4g). This result confirmed that when the reaction was performed in the co-presence of Δ^1^-pyrroline-2-carboxylate reductase^15^, L-proline was produced in a time-dependent manner (Fig. 4b). Collectively, these results suggest that the PaLhpI protein catalyzes (only) the irreversible dehydration reaction of *cis*-3-hydroxy-L-proline to Δ^1^-pyrroline-2-carboxylate via a putative Δ^2^-pyrroline-2-carboxylate intermediate (Fig. 1c). Since *cis*-3-hydroxy-L-proline possesses the hydroxyl group and proton to be eliminated at the same “*anti*” positions as Acn enzymes, it is likely that *trans*-3-hydroxy-L-proline (“*syn*” positions) is not a substrate for this protein.

In order to investigate substrate specificity in more detail, we prepared a unique 2,3-*cis*-3,4-*cis*-3,4-dihydroxy-L-proline [(2 *S*,3 *S*,4 *R*)-3,4-dihydroxyproline] from *cis*-3-hydroxy-L-proline by L-proline *cis*-4-hydroxylase from *S. meliloti*^30^. This proline derivative was an additional substrate for PaLhpI, whereas specific activity (0.130 μmol·min^−1^·mg^−1^), estimated by HPLC analysis, was ~300-fold lower than that of *cis*-3-hydroxy-L-proline (Fig. 4c). Although the ^1^H NMR analysis did not identify the enzyme product directly (Fig. S2), *cis*-4-hydroxy-L-proline was produced by coupling with Δ^1^-pyrroline-2-carboxylate reductase (Fig. 4d); the reaction product of 2,3-*cis*-3,4-*cis*-3,4-dihydroxy-L-proline must be a Δ^1^-pyrroline-4*S*-hydroxy-2-carboxylate. Furthermore, no dehydration (degradation) was observed in L-serine, in spite of carbons 1~3 as well as the hydroxyl and amino groups being identical to those of *cis*-3-hydroxy-L-proline (Fig. S3). These results indicate that PaLhpI recognizes not only the framework and chiral selectivity of 3-hydroxyproline, but also the 4′-functional group of *cis*-3-hydroxy-L-proline.

*Labrenzia aggregata* IAM 12614 possesses the L-hydroxyproline gene cluster, which contains the *LhpJ* gene (SIAM614_RS19325) instead of the *LhpI* gene (Fig. 1d)^16^. The LhpJ protein belongs to the muconate lactonizing enzyme subclass of the enolase superfamily, distinct from the Acn superfamily, and shows the bifunctional activity of (1) the reversible 2-epimerization of *cis*-3-hydroxy-L-proline to *trans*-3-hydroxy-D-proline and (2) the irreversible dehydration of *cis*-3-hydroxy-L-proline to Δ^1^-pyrroline-2-carboxylate (Fig. 1c); the latter is homologous with the activities of hydroxyproline betaine 2-epimerase^31^ and proline betaine racemase^32^, other members in this protein superfamily. On the other hand, epimerization activity was not found in PaLhpI (Fig. 4g), possibly due to the different catalytic mechanisms of the proteins, as described below.

### Catalytic properties

**Figure 5:**
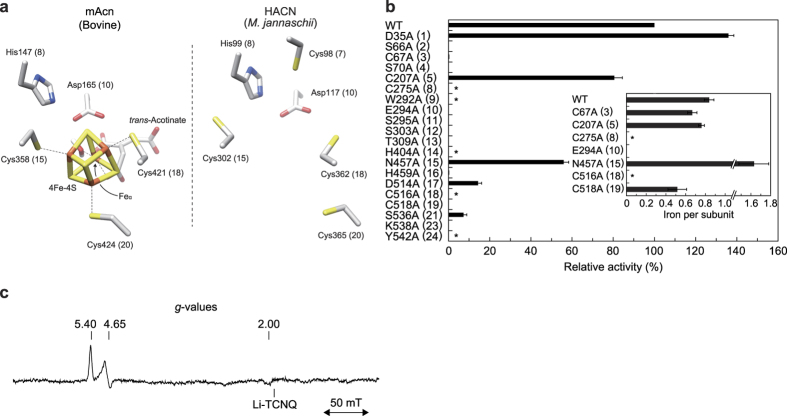
Identification of active sites. (**a**) Active site models of mAcn from bovine (PDB ID 1ACO)^19^ and ([Fe-S] cluster unbound) HACN of *M. jannaschii* (4KP2)^42^. So-called “Fe_α_” is not ligated to a cysteine residue. (**b**) Site-directed mutagenic study of PaLhpI. Numbers in parentheses correspond to the sites in Fig. 7. Relative activity values (average ± SD, *n* = 3) are expressed as percentages of the values obtained in the WT enzyme. Proteins with asterisks were not expressed in *P. putida* cells. *Inset*. Stoichiometric analysis of the iron atom. Values are the average ± SD, *n* = 3. (**c**) EPR spectra of Na_2_S_2_O_4_-reduced AtLhpI at 10 K.

In the absence of substrate under oxidative conditions, one of the iron ions, so-called “Fe_α_” (Fig. 5a), is easily lost from the [4Fe-4S] cluster in Acn enzymes, leading to the catalytically inactive [3Fe-4S] state; therefore, reconstruction (reactivation) is typically necessary under reducing conditions^33^. Purified PaLhpI was brown in color, indicating the presence of cofactor(s) (Fig. 3a), whereas inactivation was not detected after three months at −35 °C. The pH dependence of dehydration activity with *cis*-3-hydroxy-L-proline was estimated by a colorimetric method based on the reaction of 2-aminobenzaldehyde with Δ^1^-pyrroline-2-carboxylate^14^,^15^: optimum pH of 8.0–9.5 (Fig. 3c). Moreover, we developed a more conventional spectrophotometric assay method using NADPH-dependent Δ^1^-pyrroline-2-carboxylate reductase as a coupling enzyme (see Fig. 4b). Kinetic parameters with *cis*-3-hydroxy-L-proline are shown in Fig. 4h. The specific activity value, 41.3 μmol·min^−1^·mg^−1^, was similar to that obtained by HPLC.

**Figure 6:**
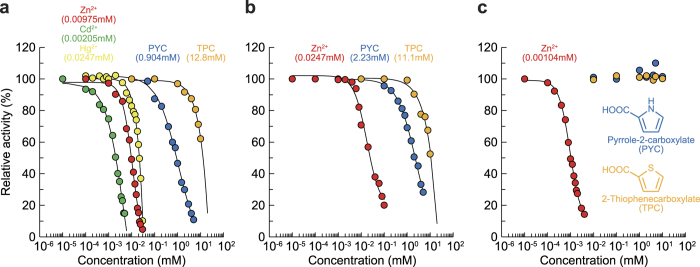
Inhibition study on *cis*-3-hydroxy-L-proline dehydratase activity. PaLhpI (**a**), AtLhpI (**b**), and TrLhpI (**c**). Relative specific activity values (average ± SD, *n* = 3) are expressed as percentages of the values obtained in the absence of an inhibitor. IC_50_ values (in parentheses) were calculated by curve fitting using ImageJ software (http://rsb.info.nih.gov/ij/).

Among the various divalent metal ions tested, only Zn^2+^, Cd^2+^, and Hg^2+^ inhibited activity (Fig. S4a), and their IC_50_ values were 9.75 × 10^−3^, 2.05 × 10^−3^, and 2.47 × 10^−2^ mM, respectively (Fig. 6a). The pattern was competitive, and the *K*_i_ value for Zn^2+^ was 1.07 × 10^−3^ mM (Fig. S4b). In the case of Acn enzymes, only mitochondrial Acn from mammals was inhibited by Zn^2+^ (*K*_i_ = 2.00 × 10^−3^ mM)^34^. Furthermore, pyrrole-2-carboxylate (and its derivative 2-thiophenecarboxylate) inhibited the activity of PaLhpI (Fig. 6a), whereas several characteristic inhibitors for Acn enzymes including *trans*-aconitate, fluorocitrate, and oxalomalate did not. Pyrrole-2-carboxylate is structurally analogous to the (putative) transition state of *cis*-3-hydroxy-L-proline, Δ^2^-pyrroline-2-carboxylate (see Fig. 1c), and its IC_50_ value is markedly higher than those of divalent metal ions: 440-fold that of Cd^2+^. Collectively, these results strongly indicate that the substrate binding pocket of the PaLhpI protein is not similar to Acn enzymes, and that binding sites for divalent metal ions and pyrrole-2-carboxylate are spatially distinct.

### Characterization of other AcnX_Type I_ enzymes

Although the bacterial *LhpI* gene is often located within the L-hydroxyproline gene cluster, there are several combinations of *trans*-4-hydroxy-L-proline and/or *trans*-3-hydroxy-L-proline metabolic genes. For example, there are two types of *cis*-4-hydroxy-D-proline dehydrogenases^13^: *P. aeruginosa* PAO1 etc., α_4_β_4_γ_4_-type enzyme encoded by *LhpB* (encoding to β-subunit), *LhpC* (α-subunit) and *LhpD* genes (γ-subunit); *Agrobacterium tumefaciens* C58 etc., homomeric-type enzyme encoded by *LhpB* gene (Fig. 1d). Furthermore, there is no sequence similarity between LhpH and LhpK proteins encoding to Δ^1^-pyrroline-2-carboxylate reductase. Although the AcnX protein was originally considered to only be present in archaea and bacteria^27^, a homology search using the Protein-BLAST program revealed that a large number of fungi possess the *LhpI* homologous gene; the ANI_1_578044 gene from *Aspergillus niger* CBS 513.88 is closely located to the putative *trans*-3-hydroxy-L-proline dehydratase gene (Fig. 1d)^14^.

Therefore, in order to elucidate the catalytic functions of other AcnX_Type I_ proteins, we enzymatically investigated Atu4683 from *A. tumefaciens* C58 (AtLhpI) and TRIREDRAFT_59073 from *Trichoderma reesei* QM6a (TrLhpI), and found 46.2% and 51.7% sequence identity with PaLhpI, respectively. The (His)_6_-tagged AtLhpI and TrLhpI proteins were expressed in *Escherichia coli*, purified, and characterized (Fig. 3a). As observed for PaLhpI, among eight proline derivatives (Fig. S1), only *cis*-3-hydroxy-L-proline was consumed in a time-dependent manner by both proteins, and similar inhibition by Zn^2+^ was observed (Fig. 6b,c). By contrast, the *k*_cat_/*K*_m_ value of TrLhpI was 316- and 83.4-fold lower than those of PaLhpI and AtLhpI, respectively (Fig. 4h). Although analysis by an amino acid analyzer suggested the reaction product of TrLhpI is Δ^1^-pyrroline-2-carboxylate (specific activity of 3.75 μmol·min^−1^·mg^−1^), the consumption rate of *cis*-3-hydroxy-L-proline by coupling assay with Δ^1^-pyrroline-2-carboxylate reductase decreased significantly (Fig. 4e,f). Furthermore, no effect of pyrrole-2-carboxylate (and 2-thiophenecarboxylate) was found on the activity (Fig. 6c). These results suggest that the fungal TrLhpI protein may well have an entirely different main (physiological) substrate from *cis*-3-hydroxy-L-proline.

### Putative active sites

**Figure 7:**
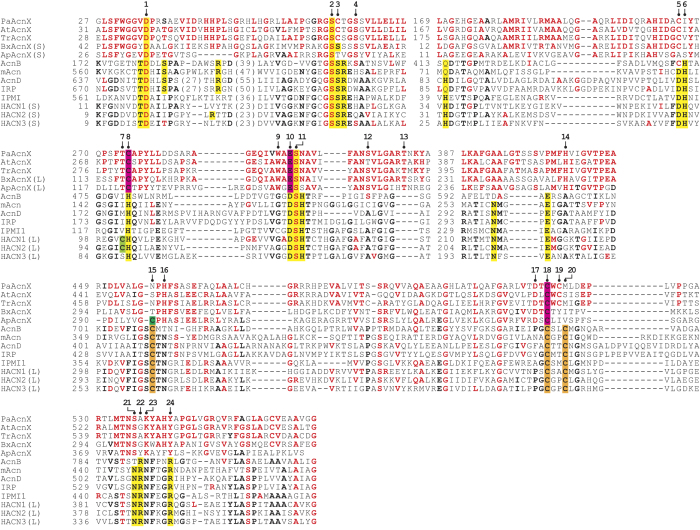
Partial multiple amino acid sequence alignments of AcnX and other Acn subfamilies. PaAcnX and AcnX from *P. aeruginosa* PAO1; AtAcnX, *A. tumefaciens* C58; TrAcnX, AcnX from *T. reesei* QM6a; BxAcnX, *Burkholderia xenovorans* LB400; ApAcnX, *Aeropyrum pernix* K1; AcnB from *E. coli*^18^; mAcn from bovine^19^; AcnD from *Shewanella oneidensis* MR-1^21^; IRP from human^20^; IPMI from *Saccharomyces cerevisiae*^26^; HACN1 and HACN2 from *M. jannaschii*^23^,^42^; HACN3 from *Pyrococcus horikoshii* OT3^43^. “S” and “L” indicate small and large subunits, respectively. Red- and black-bold letters indicate amino acids highly conserved in the AcnX subfamily and Acn enzymes, respectively. The complete alignment of the AcnX subfamily is shown in Fig. S5.

There are ~23 amino acid residues that are completely (or highly) conserved between Acn enzymes (yellow- and orange-shadowed letters in Fig. 7), implying similar structural organization and catalytic mechanisms^35^. However, most of these residues had no readily detectable counterpart in AcnX_Type I_ proteins. On the other hand, several amino acid residues were completely (or highly) conserved within the AcnX subfamily only (Fig. 7 and Fig. S5), and some of them may be located around the active sites of Acn enzymes. Based on these insights, we selected thirty amino acid residues at sites 1~4, 9, 11~14, 17, and 21~23 for a site-directed mutagenic study, and constructed each alanine mutant of PaLhpI (Fig. 5b). Among them, only D35A (136%), C207A (80.5%), and N457A (55.9%) mutants showed similar activity to the wild-type (WT) enzyme, whereas C275A, W292A, H404A, C516A, and Y542A mutants were not expressed in host cells, S66A, S70A, E294A, S295A, S303A, T309A, C518A, and K538A mutants were inactive, and C68A (0.4%), H459A (0.4%), D523A (14.4%) and S545A (7.3%) mutants decreased the activity significantly (the values in the parentheses are the expression of specific activity relative to the WT enzyme), suggesting their potential role(s) in catalysis and/or structure folding. Among them, Ser66 at site 2 may play a role in abstracting a proton from the Cα of *cis*-3-hydroxy-L-proline as a catalytic base, as in Acn enzymes^33^. However, in contrast to Cys275 of PaLhpI, mutation of histidine of Acn enzymes at site 8 does not markedly change activity levels from those of the WT enzyme^36^, probably caused by the lack of a direct ligand for [4Fe-4S] cluster, as described below.

### Putative iron binding sites

Inhibition by the Hg^2+^ and Cd^2+^ of LhpI protein(s) is observed in many proteins that possess a cysteine residue(s) as the active site: furthermore, an incubation with Hg^2+^ results in irreversible inactivation (Fig. S4c). In the case of Acn enzymes, three iron ions of the [4Fe-4S] cluster were coordinated by three conserved cysteine residues (orange-shadowed letters at sites 15, 18, and 20 in Fig. 5a and Fig. 7)^33^, among which PaLhpI possessed only one equivalent residue to site 18 (Cys516). Therefore, as other potential ligand(s), we selected Cys207, Cys275, Glu294, Asn457, and Cys518 at sites 5, 8, 10, 15, and 19, the corresponding residues of the Acn enzymes that were located around the [4Fe-4S] cluster, and measured the iron content in each alanine mutant by a colorimetric assay. As results, WT, C207A, E294A, N457A, and C518A contained 0.84, 0.76, 0.01>, 1.63, and 0.52 iron equivalents per (monomeric) protein (Fig. 5b): Cys275A and C516A mutants were not expressed in host cells, as described above. These findings indicate that PaLhpI contains an iron ion (but not [4Fe-4S] cluster) that is necessary for catalysis, and that Cys275, Glu294, and Cys516 at sites 8, 10, and 18 may be important for substrate binding (pink-shadowed letters). Complete conservation at these sites in the AcnX subfamily appears to support this hypothesis (Fig. S5). It is noteworthy that in the catalytic mechanism of the enolase type of *cis*-3-hydroxy-L-proline dehydratase (LhpJ), an enolate anion intermediate is stabilized by an essential Mg^2+^ ligated by two tightly conserved aspartate residues and one glutamate residue, and two lysine residues function as a basic and/or acidic catalyst^16^. Therefore, although AcnX_Type I_ has no evolutionary relationship with the enolase type enzyme, the catalytic mechanism including metal ion binding may be more similar than those of Acn enzymes.

### Non-heme iron analysis

In order to examine the nature of non-heme iron in more detail, electron paramagnetic resonance (EPR) analysis was performed using AtLhpI. AtLhpI treated with dithionite (Na_2_S_2_O_4_) exhibited EPR signals at *g* = 5.40, 4.65, and 2.00 (Fig. 5c), whereas reduced rubredoxin, a typical mononuclear iron-sulfur protein, was EPR-silent. This EPR spectrum is markedly different from those of other reduced iron-sulfur clusters, such as [2Fe-2S] ferredoxin, the [2Fe-2S] Rieske center, [3Fe-4S] ferredoxin, [3Fe-4S] Acn enzymes, and [4Fe-4S] ferredoxin, the EPR signals of which were observed in the *g* ≈ 2 region^37^. Furthermore, it is interesting to note that the observed *g*-values of AtLhp1 were not a typical high-spin (*S* = 5/2) electronic ground state. Similar EPR spectra have been reported for cytochrome *c*’ from some photosynthetic bacteria, the electronic ground states of which are interpreted as quantum mechanical admixtures of an intermediate-spin state (*S* = 3/2) and high-spin state (*S* = 5/2)^38^,^39^. The *S* = 3/2 and *S* = 5/2 components at the ground state of the iron center in AtLhp1 are estimated to be 49 and 51%, respectively, from observed *g*-values (5.40 and 4.65). Although the electronic ground state of iron is still ambiguous, these results suggest that the iron site in AtLhpI is an unprecedented mononuclear Fe(III) center that has a low redox potential that is resistant to the reduction of dithionite, but not an iron-sulfur cluster. In rubredoxin, Fe(III) is coordinated to four cysteinyl sulfurs occurring on two Cys-X_2_-Cys segments or one Cys-X_2_-Cys and one Cys-Cys^40^. By contrast, in order to coordinate the Fe(II) center, “cupin superfamily” proteins have a conserved motif, in which no cysteine is contained as a ligand: His-X_2_-[His/Asp]-X_4_-Glu-X_n_-His^41^. These specific motifs are not found in the sequences of AcnX enzymes (Fig. S5).

AcnX_Type IIb_ proteins possess two homologous cysteine residues, not only at site 18 but also site 15, with Acn enzymes (green-shadowed cysteine in Fig. 7 and Fig. S5). On the other hand, in the HACN subfamily, only enzymes from methanogenic archaea, including *Methanococcus jannaschii*, have the “fourth cysteine” at site 7 (light-green shadowed cysteine), which may be located near a sulfur atom in the [4Fe-4S] cluster; however, its role is currently unclear (Fig. 5a)^42^,^43^. Indeed, we have already found that purified AcnX_Type IIb_ protein (from *Aeropyrum pernix* K1) also contains iron ion(s), although functional annotation is in progress (unpublished). Therefore, the EPR analysis of AcnX_Type IIb_ protein would be useful for further understanding of the unique binding mode of Fe(III) of AcnX subfamily, and identifying which of the [4Fe-4S] cluster, or Fe(III), common ancestors of the Acn superfamily possessed.

### Degradation pathway of L-hydroxyproline(s) in *P. aeruginosa* PAO1

**Figure 8:**
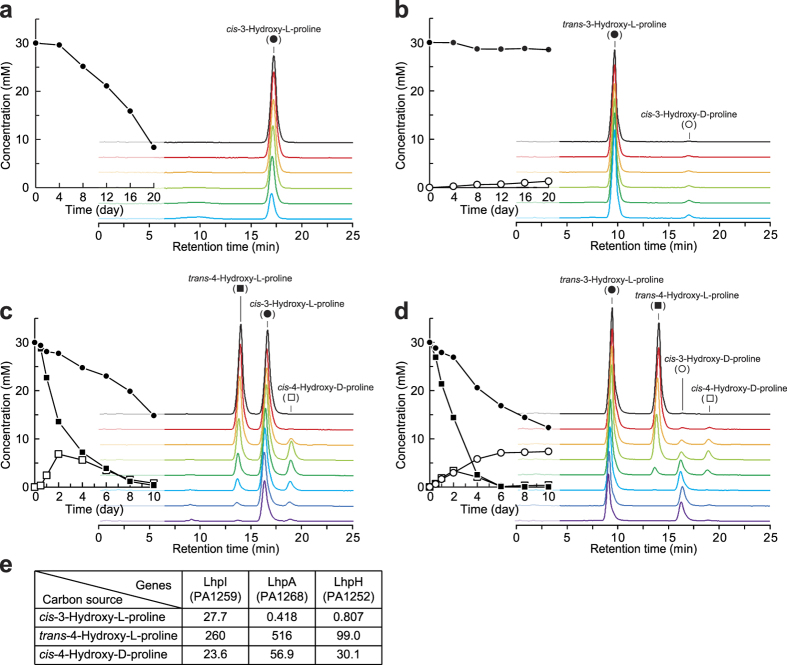
L-Hydroxyproline metabolism in *P. aeruginosa* PAO1. L-Hydroxyproline degradation when *P. aeruginosa* PAO1 was cultivated on *cis*-3-hydroxy-L-proline (**a**), *trans*-3-hydroxy-L-proline (**b**), *cis*-3-hydroxy-L-proline + *trans*-4-hydroxy-L-proline (**c**), and *trans*-3-hydroxy-L-proline + *trans*-4-hydroxy-L-proline (**d**) as a sole carbon source(s). Concentrations of L-hydroxyproline in medium were measured using an amino acid analyzer. Three independent experiments were performed, and the typical results obtained are shown. (**e**) Transcriptional analysis. Expression profiles in cells grown with *cis*-3-hydroxy-L-proline, *trans*-4-hydroxy-L-proline, and *cis*-4-hydroxy-D-proline compared to those in cells grown with L-proline as a sole carbon source. Numbers indicate relative expression levels that were measured by qRT-PCR.

To the best of our knowledge, *cis*-3-hydroxy-L-proline has only been found as one of the building blocks of telomycin, a peptide antibiotic produced by *Streptomyces canus* C159^7^,^8^, and these bacteria may also hydroxylate free L-proline to *cis*-3-hydroxy-L-proline enzymatically^6^. Since the amount of *cis*-3-hydroxy-L-proline in nature is markedly less than that of *trans*-4-hydroxy-L-proline, the possibility of catabolism by (micro)organisms had not previously been considered. In order to estimate the potential metabolism of L-hydroxyproline, *P. aeruginosa* PAO1 was cultivated in minimal medium supplemented with L-proline, *trans*-4-hydroxy-L-proline, *trans*-3-hydroxy-L-proline, or *cis*-3-hydroxy-L-proline as the sole carbon source. Measurements of concentration by an amino acid analyzer revealed that *cis*-3-hydroxy-L-proline (but not *trans*-3-hydroxy-L-proline) was consumed in a time-dependent manner: 72% degradation after 20 days (Fig. 8a,b). Furthermore, in comparisons with L-proline in a quantitative real-time PCR (qRT-PCR) analysis, the *PaLhpI* gene was induced not only by *trans*-4-hydroxy-L-proline and *cis*-4-hydroxy-D-proline, but also by *cis*-3-hydroxy-L-proline, whereas the *PaLhpA* and *PaLhpH* genes were only induced by *trans*-4-hydroxy-L-proline and *cis*-4-hydroxy-D-proline (Fig. 8e). Since the co-presence of *trans*-4-hydroxy-L-proline clearly enhanced the metabolism of *cis*-3-hydroxy-L-proline (Fig. 8c), slow degradation may be due to the lack of significant induction of the *PaLhpH* gene. Similarly, *Starkeya novella* DSM 506, which possesses the *LhpJ* gene (Fig. 1d), may utilize *cis*-3-hydroxy-L-proline as a carbon source, and transcription is up-regulated by *cis*-3-hydroxy-L-proline (and *trans*-4-hydroxy-L-proline)^16^. Although the co-presence of *trans*-4-hydroxy-L-proline also resulted in the degradation of *trans*-3-hydroxy-L-proline, half of the *trans*-3-hydroxy-L-proline consumed accumulated as a *cis*-3-hydroxy-D-proline (Fig. 8d), indicating the presence of *trans*-3-hydroxy-L-proline metabolic enzyme(s). We herein demonstrated that *PaLhpL* gene (PA1255; Fig. 1d) in the L-hydroxyproline gene cluster encodes a bifunctional dehydratase and 2-epimerase toward *trans*-3-hydroxy-L-proline as the leading candidate (Supplementary Discussion and Fig. S6).

Overall, the L-hydroxyproline gene cluster from microorganisms is often related not only to the metabolism of *trans*-4-hydroxy-L-proline, but also to that of *cis*-3-hydroxy-L-proline and *trans*-3-hydroxy-L-proline, suggesting that the origin of these free L-hydroxyproline compounds in nature is the degradation of several peptide antibiotics, such as telomycin (containing *cis*-3-hydroxy-L-proline and *trans*-3-hydroxy-L-proline)^7^,^8^, microcolin A (*cis*-4-hydroxy-L-proline)^9^, pneumocandins (*trans*-4-hydroxy-L-proline and *trans*-3-hydroxy-L-proline)^10^, and etamycin (*cis*-4-hydroxy-D-proline)^11^, rather than collagen.

## Conclusion

We herein showed that LhpI protein catalyzes the dehydration of *cis*-3-hydroxy-L-proline to Δ^1^-pyrroline-2-carboxylate: over 10 years after the discovery of AcnX (subfamily) in Acn superfamily^27^, the functional characterization can now be done successfully. The gene context in the bacterial genome was of considerable help in estimating the potential substrate, rather than the amino acid sequence similarity. Although *cis*-3-hydroxy-L-proline is believed to hardly exist in nature, our findings may indicate the significant physiological role in (micro)organisms, similar to *trans*-3-hydroxy-L-proline^14^,^15^. Until now, the Acn superfamily (enzymes) was a typical example suitable for a recruitment hypothesis of enzyme evolution proposed by Jensen (1976)^44^; gene duplication of multi-specific enzymes containing a [4Fe-4S] cluster, followed by narrowing of substrate specificity. Moreover, this functional annotation of a Fe(III)-containing AcnX protein must promote interest in common ancestors of the Acn superfamily, including the nature of the iron-sulfur cluster.

## Methods

### Plasmid construction for the expression of recombinant proteins

The primer sequences used in this study are shown in Table S2. *PaLhpI, TrLhpI*, and *AtLhpI* genes were amplified by PCR using primers containing appropriate restriction enzyme sites at the 5′- and 3′-ends and the genome DNA of *P. aeruginosa* PAO1, *T. reesei* QM6a, or *A. tumefaciens* C58 as a template. Each amplified DNA fragment was introduced into BamHI-HindIII sites in pQE-80L (Qiagen), a plasmid vector for conferring an N-terminal (His)_6_ tag on the proteins expressed, in order to obtain pQE/PaLhpI, pQE/TrLhpI, and pQE/AtLhpI, respectively. Regarding the expression of the *PaLhpI* gene in *P. putida* cells, a DNA fragment of the (His)_6_-PaLhpI-*t*_0_ terminator was amplified by PCR using pQE/PaLhpI as a template, and introduced into the SalI-EcoRI sites in pUCP26KmAhpC_p_^45^ in order to obtain pUCP/PaLhpI. A site-directed mutation was introduced into the *PaLhpI* gene by sequential steps of PCR using sense and antisense primers and pUCP/PaLhpI as a template.

### Expression and purification of the recombinant protein

*P. putida* KT2442-oxyR1^46^ harboring pUCP/PaLhpI was grown at 30 °C overnight in LB medium containing kanamycin (50 mg/liter), whereas *E. coli* strain DH5α harboring pQE/TrLhpI or pQE/AtLhpI was grown at 37 °C to a turbidity of 0.6 at 600 nm in Super broth medium containing ampicillin (50 mg/liter). After the addition of 1 mM isopropyl-β-D-thiogalactopyranoside (IPTG), the culture was grown at 18 °C for a further 18 h in order to induce the expression of the (His)_6_-tagged protein. Grown cells were harvested, suspended in Buffer A (50 mM sodium phosphate buffer (pH 8.0) containing 300 mM NaCl and 10 mM imidazole), disrupted by sonication, and then centrifuged. The supernatant was loaded onto a Ni-NTA Superflow column (Qiagen) equilibrated with Buffer A linked to the BioAssist eZ system (TOSOH). The column was washed with Buffer B (50 mM sodium phosphate buffer (pH 8.0) containing 300 mM NaCl, 10% (v/v) glycerol, and 50 mM imidazole). The enzymes were then eluted with Buffer C (pH 8.0, as Buffer B but containing 250 mM instead of 50 mM imidazole), concentrated by ultrafiltration, dialyzed against 50 mM Tris-HCl buffer (pH 8.0) containing 50% (v/v) glycerol, and stored at –35 °C until use. The native molecular mass of the recombinant protein was estimated by gel filtration using a HiLoad 16/60 Superdex 200 pg column (GE Healthcare) equilibrated with 50 mM Tris-HCl buffer (pH 8.0). A high-molecular-weight gel filtration calibration kit (GE Healthcare) was used for molecular markers. Protein concentrations were measured by the method of Lowry *et al*.^47^ with bovine serum albumin as the standard.

### Screening of the proline derivative library for the LhpI protein

The purified PaLhpI protein (5 μg) was added to 50 mM Tris-HCl buffer (pH 8.0) (1 ml) containing 10 mM substrate (Fig. S1), and incubated at 30 °C. After varying the incubation times, the enzyme reaction was stopped by briefly incubating at −80 °C. Substrate consumption was estimated by HPLC using an Ultron ES-CD chiral separation column (150 × 2.0 mm, Shinwa Chemical Industries)^48^ and/or Hitachi L-8900 PH Amino Acid Analyzer (Hitachi, Tokyo, Japan).

### Enzyme assay

Unless noted otherwise, *cis*-3-hydroxy-L-proline dehydratase was assayed spectrophotometrically in the coupling system with Δ^1^-pyrroline-2-carboxylate reductase (LhpH protein from *P. aeruginosa* PAO1)^15^. The reaction mixture consisted of 50 mM Tris-HCl (pH 8.0), 0.15 mM NADPH, and purified Δ^1^-pyrroline-2-carboxylate reductase (10 μg). The reaction was initiated by the addition of 100 mM *cis*-3-hydroxy-L-proline (100 μl) with a final reaction volume of 1 ml. One unit of enzyme activity refers to 1 μmol NADPH consumed/min. *K*_m_ and *k*_cat_ values were calculated by a Lineweaver-Burk plot. The enzyme was alternatively assayed by a colorimetric method based on the reaction of 2-aminobenzaldehyde with Δ^1^-pyrroline-2-carboxylate, which yields a yellow reaction product^13^,^14^. This method was used to clarify the optimum pH for enzyme activity. Potential Acn activity in PaLhpI protein was measured spectrophotometrically by monitoring the change of absorption at 240 nm derived from unsaturated *cis*-aconitate^49^.

### Identification of reaction products

In order to remove glycerol in the storage buffer, purified PaLhpI was dialyzed at 4 °C overnight in 50 mM K_2_HPO_4_/KH_2_PO_4_ buffer (pH 7.0), lyophilized, and solvated in D_2_O (600 μl) containing 20 mM *cis*-3-hydroxy-L-proline or 2,3-*cis*-3,4-*cis*-3,4-dihydroxy-L-proline. NMR spectra were recorded at 25 °C on a JEOL JNM-EC400 NMR spectrometer (JEOL Ltd., Tokyo, Japan) operating at 400 MHz. 2,2-Dimethyl-2-silapentane-5-sulfonate was used as an internal standard. 2,3-*cis*-3,4-*cis*-3,4-Dihydroxy-L-proline was enzymatically synthesized from *cis*-3-hydroxy-L-proline by L-proline *cis*-4-hydroxylase from *S. meliloti*, as described previously^30^.

### Non-heme iron analysis

The content of non-heme iron was assessed using a Metallo Assay kit (AKJ Global Technology, Japan). The (potential) iron-sulfur cluster was analyzed by electron paramagnetic resonance (EPR) using a JEOL TE-300 X-band spectrometer operating with a 100-kHz field modulation. A temperature-dependent analysis was performed in the range of 10 to 40 K using a LTR-3 liquid helium cryostat (Air Products). The purified AtLhpI protein (~40 mg/ml) was prepared in 50 mM Tris-HCl (pH8.0) containing 50% (v/v) glycerol for this purpose, and treated with a 10-fold excess amount of Na_2_S_2_O_4_ under a nitrogen atmosphere. EPR spectra were recorded using the following representative conditions; microwave frequency; 8.9995 GHz, microwave power; 5.0 mW, 100 kHz modulation magnitude; 0.63 mT, center field; 280 ± 250 mT, sweep time; 4.0 min, time constant; 0.1 sec, and receiver amplitude; 500. In the present study, *g*-values were evaluated based on the *g*-value of the Li-salt of tetracyanoquinodimethane (2.0025) as an external standard. The magnetic field strength of EPR spectra was calibrated using the hyperfine coupling constant (8.69 mT) of the Mn(II) ion doped in MgO powder.

### Growth and gene expression analysis of *P. aeruginosa* PAO1

*P. aeruginosa* PAO1 was cultured aerobically with vigorous shaking at 30 °C in 5 ml of minimal medium^15^ supplemented with a 30 mM carbon source(s). The concentration of L-hydroxyproline in medium after varying cultivation times was analyzed using an amino acid analyzer (L-8900; Hitachi, Tokyo, Japan). The preparation of RNA samples and one-step real-time RT-PCR were performed as described previously^50^. The primers used for RT-PCR are listed in Table S2. The PA0393 gene encoding pyrroline-5-carboxylate reductase was used as an internal control.

### Sequence comparison

Protein sequences were analyzed using the Protein-BLAST and Clustal W programs distributed by DDBJ (DNA Data Bank of Japan).

## Additional Information

**How to cite this article**: Watanabe, S. *et al*. Functional characterization of aconitase X as a *cis*-3-hydroxy-L-proline dehydratase. *Sci. Rep.* **6**, 38720; doi: 10.1038/srep38720 (2016).

**Publisher's note:** Springer Nature remains neutral with regard to jurisdictional claims in published maps and institutional affiliations.

## Supplementary Material

Supplementary Information

## Figures and Tables

**Figure 2:**
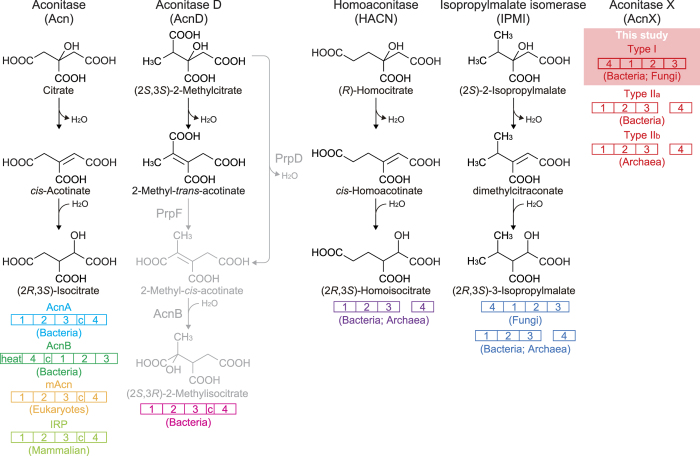
Schematic reactions of four members of the Acn superfamily. A linear representation of the sequential domain arrangement of six phylogenetic subfamilies is also included. “c” is the connector domain, and “heat” is a protein-protein interaction domain found only in AcnB^18^. In propionate metabolism in bacteria^21^,^51^, (2*S*,3*S*)-2-methylcitrate is converted to 2-methyl-*cis*-acotinate through two routes: (1) sequential reactions by AcnD and PrpF via 2-methyl-*trans*-acotinate; (2) reaction by a single protein, PrpD (gray-colored routes). 2-Methyl-*cis*-acotinate is subsequently metabolized to (2*S*,3*R*)-2-methylisocitrate by AcnB. The AcnX subfamily is further classified into three groups, among which AcnX_Type I_, consisting of a single polypeptide from bacteria, was functionally characterized in this study.
