# Supplementary material for: Functional characterization of aconitase X as a cis-3-hydroxy-L-proline dehydratase
Source: Sci Rep. 2016 Dec 8;6:38720. doi: 10.1038/srep38720 (PMC5144071; doi:10.1038/srep38720)
Supplement: Supplementary Information [file srep38720-s1.pdf]

Supplementary information

Functional characterization of aconitase X as a *cis*-3-hydroxy-L-proline dehydratase.

Seiya Watanabe[1,2,3], Kunihiko Tajima[4], Satoshi Fujii[5], Fumiyasu Fukumori[6], Ryotaro Hara[7], Rio Fukuda[2], Mao Miyazaki[2], Kuniki Kino[7,8], Yasuo Watanabe[1,2]

[1] Department of Bioscience, Graduate School of Agriculture, Ehime University, 3-5-7 Tarumi, Matsuyama, Ehime 790-8566, Japan
[2] Faculty of Agriculture, Ehime University, 3-5-7 Tarumi, Matsuyama, Ehime 790-8566, Japan
[3] Center for Marine Environmental Studies (CMES), Ehime University, 2-5 Bunkyo-cho, Matsuyama, Ehime 790-8577, Japan
[4] Department of Bio-molecular Engineering, Graduate School of Science and Technology, Kyoto Institute of Technology, Matsugasaki, Sakyo-ku, Kyoto 606-8585, Japan
[5] Faculty of Frontiers of Innovative Research in Science and Technology (FIRST), Konan University, 7-1-20 Minatojima-minamimachi, Chuo-ku, Kobe, Hyogo 650-0047, Japan
[6] Faculty of Food and Nutritional Sciences, Toyo University, 1-1-1 Izumino, Itakura-machi, Ora-gun, Gunma 374-0193, Japan
[7] Research Institute for Science and Engineering, Waseda University, 3-4-1 Ohkubo, Shinjuku-ku, Tokyo 169-8555, Japan
[8] Department of Applied Chemistry, Faculty of Science and Engineering, Waseda University, 3-4-1 Ohkubo, Shinjuku-ku, Tokyo 169-8555, Japan

Correspondence and requests for materials should be addressed to S.W. (e-mail: irab@agr.ehime-u.ac.jp).

Supplementary Discussion

Characterization of PaLhpL protein. The recombinant $(His)_6$-tagged PaLhpL protein was successfully expressed in *P. putida* cells, and purified to homogeneity using a nickel-chelating affinity column (Fig. S6a). In the first approach for functional analysis, eight proline derivatives (10 mM; Fig. S1) were tested as substrates in Tris-HCl buffer (pH 8.0) without additives. Among them, only *trans*-3-hydroxy-L-proline was consumed in a time-dependent manner, and the reaction product was identified as *cis*-3-hydroxy-D-proline by an amino acid analyzer (Fig. S6b). By contrast, when *trans*-3-hydroxy-L-proline was incubated with PaLhpL and PaLhpH, not only *cis*-3-hydroxy-D-proline but also L-proline was detected (Fig. S6c), suggesting bifunctional dehydratase and 2-epimerase activities toward *trans*-3-hydroxy-L-proline. The k_{cat}/K_m value of the dehydration reaction was estimated to be 1.23 $mM^{-1}\cdot min^{-1}$, which was ~6000-fold lower than that of PaLhpI toward *cis*-3-hydroxy-L-proline (Fig. S6d and Fig. 4f).

PaLhpL protein belongs to the proline racemase superfamily, which includes the archetypical proline racemase, hydroxyproline 2-epimerase, and *trans*-3-hydroxy-L-proline dehydratase; the former two are involved in L-hydroxyproline metabolism in organisms, as described in “Introduction” (Fig. 1a, b and Table S1). Until now, these enzymes had been classified into four types based on two specific residues at the active sites: Cys-Cys type, proline racemase and hydroxyproline 2-epimerase; Cys-Thr or Ser-Thr type, *trans*-3-hydroxy-L-proline dehydratase; Ser-Cys type, function unknown. In this regard, there are two types of LhpL protein, Ser-Cys or Ser-Thr, and PaLhpL protein belongs to the former. Therefore, it is likely that both types of LhpL protein commonly function as a *trans*-3-hydroxy-L-proline dehydratase.

Reference

1. Visser, W. F., Verhoeven-Duif, N. M. & de Koning, T. J. Identification of a human *trans*-3-hydroxy-L-proline dehydratase, the first characterized member of a novel family of proline racemase-like enzymes. *J. Biol. Chem.* **287,** 21654-21662 (2012).

Table S1. List of enzymes related to L-hydroxyproline metabolism in organisms.

Metabolism	Enzyme	EC number	Gene
trans-4-Hydroxy-L-proline (mammals)	L-Hydroxyproline oxidase	1.5.-.-	*PRODH*, *PRODH2*
	Δ^1-Pyrroline-3-hydroxy-5-carboxylate dehydrogenase	1.5.1.12	*ALDH4A1*
	4-Hydroxyglutamate transaminase	2.6.1.23	*GOT1*
	4-Hydroxy-2-oxoglutarate aldolase	4.1.3.16	*HOGA1*
trans-4-Hydroxy-L-proline (bacteria)	Hydroxyproline 2-epimerase	5.1.1.8	*LhpA*
	cis-4-Hydroxy-D-proline dehydrogenase	1.5.99.-	*LhpB* or *LhpBCD*
	Δ^1-Pyrroline-4*R*-hydroxy-2-carboxylate deaminase	3.5.4.22	*LhpE*
	α-Ketoglutaric semialdehyde dehydrogenase	1.2.1.26	*LhpF*
trans-3-Hydroxy-L-proline (mammals and bacteria)	*trans*-3-Hydroxy-L-proline dehydratase	4.2.1.77	*LhpG*
	Δ^1-Pyrroline-2-carboxylate reductase	1.5.1.1	*LhpH* or *LhpK*
cis-3-Hydroxy-L-proline (bacteria)	*cis*-3-Hydroxy-L-proline dehydratase	4.2.1.-	*LhpJ*
	Δ^1-Pyrroline-2-carboxylate reductase	1.5.1.1	*LhpH* or *LhpK*

Table S2. Primers used in this study.

Primer	Sequence*
Cloning of the *PaLhpI* gene into pQE-80L	
P1 (BamHI)	5′-catggatccAAGCACGCACACCTCATCGTTCCCCGTACC-3′
P2 (HindIII)	5′-attaagctTCAGCAGTCCTCGCTCAACCAGGCGGGC-3′
Cloning of the *PaLhpG* gene into pQE-80L	
P3 (BamHI)	5′-catggatccCGTTCGCAGCGGATCGTCCACATCGTCAGTTGCC-3′
P4 (HindIII)	5′-attaagctTCAGCAGTGGCCGCCGGGCCAGGTGTCGGACAGC-3′
$(His)_6$-PaLhpI and $(His)_6$-PaLhpG genes in $pUCP26KmAhpC_p$	
P5 (XhoI)	5′-ccatctcgagCACCATCACCATCACCATGGATCC-3′
P6 (MfeI)	5′-gcttcaattgTCACCAATAAAAAACGCCCGGC-3′
Cloning of the *TrLhpI* gene into pQE-80L	
P7 (BamHI)	5′-catggatccCTTCCCAGAAACTCTCATTACAAGGGAACC-3′
P8 (HindIII)	5′-attaagctTCACCTCACACCTGCTCCTTTCGGTATAC-3′
Cloning of the *AtLhpI* gene into pQE-80L	
P9 (BamHI)	5′-catggaTCcTCTGCCGTTTCAACCACAGCCGCTCCCG-3′
P10 (HindIII)	5′-attaagctTCAGGAAAGCCAGACAGGAAGGCGGGATAC-3′
Site-directed mutagenesis in the *PaLhpI* gene§	
P11 (D35A)	5′-GGGGCGGCGTCGCCCCGCGCAGCG-3′
P12 (S66A)	5′-GGTGGGCGCGGCGCCTGTACCGGC-3′
P13 (C67A)	5′-GGGCGCGGCTCCGCCACCGGCAGCAGC-3′
P14 (S70A)	5′-CCTGTACCGGCGCCAGCGTACTGC-3′
P15 (C207A)	5′-GCACATCGACGCTGCCATCTACACCGGC-3′
P16 (C275A)	5′-CCCAGCTTCACCGCCGCGCCCTACCTGC-3′
P17 (W292A)	5′-GAACAGATCGTCGCGGCCGAGTCCAAC-3′
P18 (E294A)	5′-CGTCTGGGCCGCGTCCAACGCGG-3′
P19 (S295A)	5′-GTCTGGGCCGAGGCCAACGCGGTG-3′
P20 (S303A)	5′-CTCTTCGCCAACGCCGTGCTCGGCGC-3′
P21 (T309A)	5′-CTCGGCGCACGCGCCAACAAGTACG-3′
P22 (H404A)	5′-GTGCCGATGTTCGCCGTCATCGGCG-3′

*Lower case letters indicate additional bases for introducing the digestion sites of restriction enzymes in parentheses.
§Only sense primers are shown. Underlining indicates mutated regions.

Table S2. Primers used in this study (continued).

Primer	Sequence *
P23 (N457A)	5′-GGTGGCGCTGGGCGCCCCGCACTTCTCCG-3′
P24 (H459A)	5′-GCTGGGCAACCCGGCCTTCTCCGCCAGC-3′
P25 (D514A)	5′-GCTGGTCACCGCCACCTGCTGGTG-3′
P26 (C516A)	5′-GGTCACCGACACCGCCTGGTGCATGCTCG-3′
P27 (C518A)	5′-CGACACCTGCTGGGCCATGCTCGACGAACC-3′
P28 (S536A)	5′-CTGATGACCAACGCCGCCAAGTATG-3′
P29 (K538A)	5′-CCAACTCCGCCGCGTATGCCCACTAC-3′
P30 (Y542A)	5′-CAAGTATGCCCACGCCGCCCCCGGAC-3′
qRT-PCR of the PA0393 gene	
P31 (F)†	5′-GCGAAGATCGCCGGCGAGTT-3′
P32 (R)	5′-CTGCGGCTTGACCGACAGGA-3′
qRT-PCR of the *PaLhpA* gene	
P33 (F)	5′-GCCAGGGCGACATGGCTGA-3′
P34 (R)	5′-CGCCGACCAGTACGTCGCT-3′
qRT-PCR of the *PaLhpI* gene	
P35 (F)	5′-CACCGACCGGCCTGAGCTT-3′
P36 (R)	5′-GTGTAGATGGCGGCCGCTGA-3′
qRT-PCR of the *PaLhpH* gene	
P37 (F)	5′-TTCAGCGAAGCCCATGTACAGG-3′
P38 (R)	5′-AACCGCCAGATACCGTGGGA-3′

†F and R indicate forward and reverse primers, respectively.

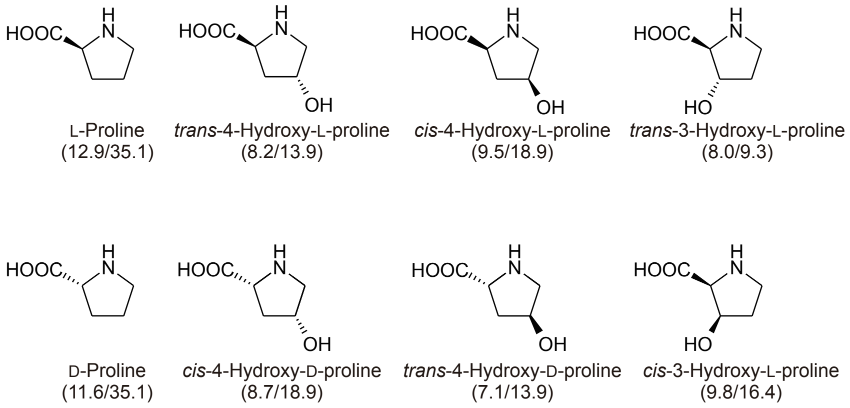

Figure S1. Library of proline derivatives tested as substrates for the PaLhpI protein. Values in parentheses indicate the elution times (min) on HPLC using a chiral separation column and Hitachi L-8900 PH Amino Acid Analyzer, respectively.

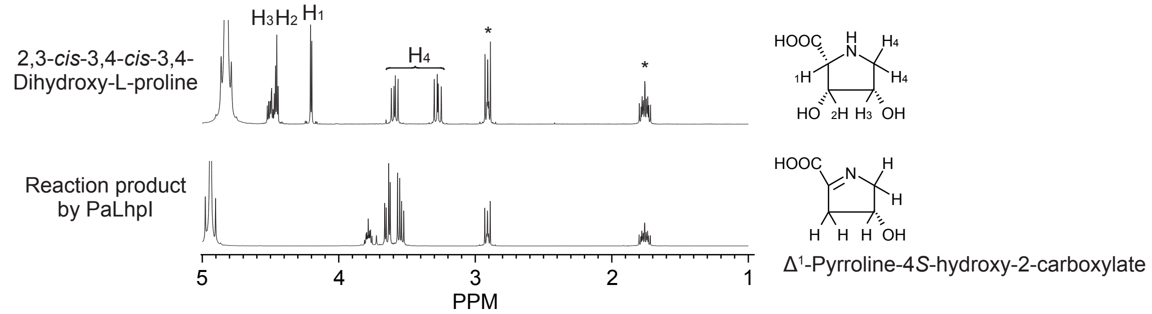

Figure S2. ^{1}H NMR spectra of 2,3-*cis*-3,4-*cis*-dihydroxy-L-proline and the reaction product by PaLhpI. The putative product is Δ^1-pyrroline-4*S*-hydroxy-2-carboxylate (inset), which is commercially unavailable.

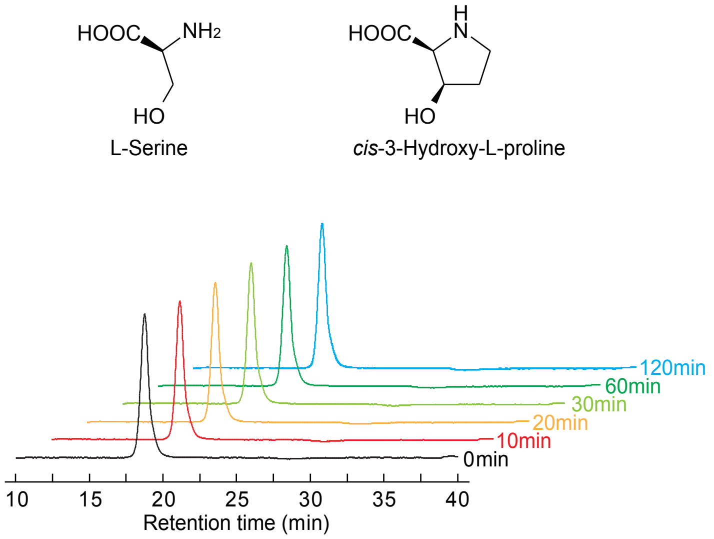

Figure S3. Estimation of dehydration activity toward L-serine by PaLhpI. A reaction mixture consisting of 50 mM Tris-HCl (pH8.0) and 10 mM L-serine was incubated for the indicated time, and analyzed by an amino acid analyzer.

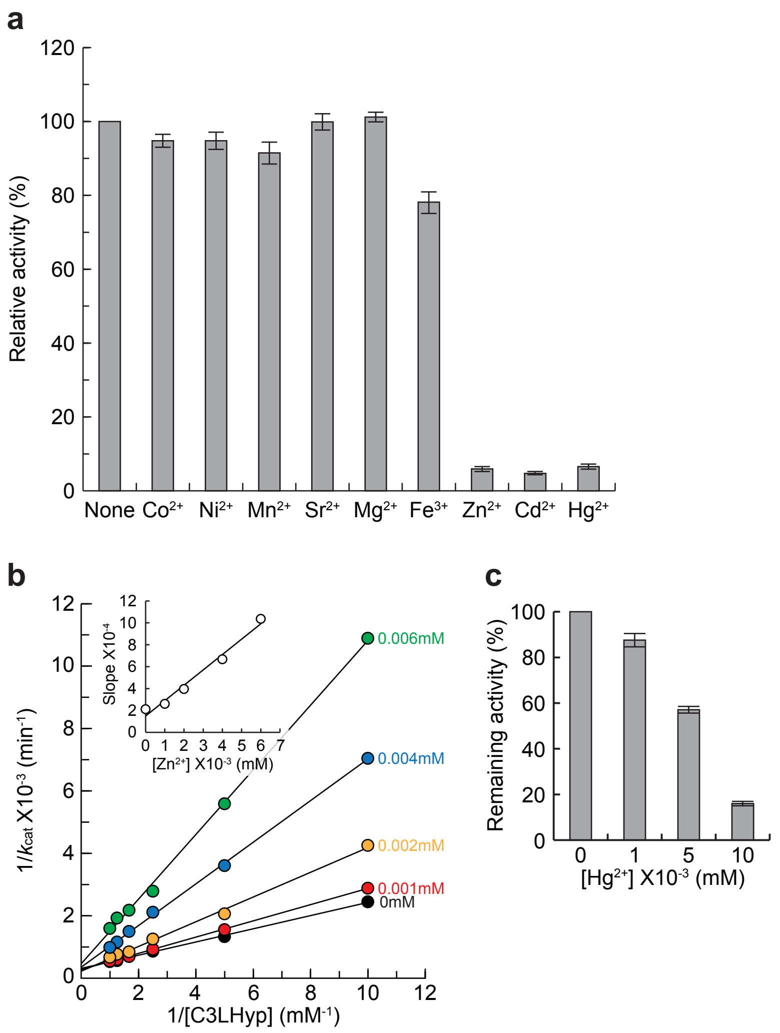

Figure S4. Effects of metal ions on the C3LHyp dehydratase of PaLhpI. (**a**) Inhibition by several metal ions. Activity was measured in the presence of 1 mM of the metal ions indicated, and expressed as values relative to maximum activity (100%) measured in the absence of metal ions. Values are the average ± SD, n = 3. (**b**) Inhibition by Zn^{2+}. C3LHyp concentrations changed with the fixed concentration of Zn^{2+} (0, 0.001, 0.002, 0.004, and 0.006 mM). The inset shows slope versus [Zn^{2+}], the K_i value of which was found to be 1.07×10^{-3} mM. (**c**) Inhibition by Hg^{2+}. The enzyme was incubated for 10 min with the indicated concentration of Hg^{2+}.

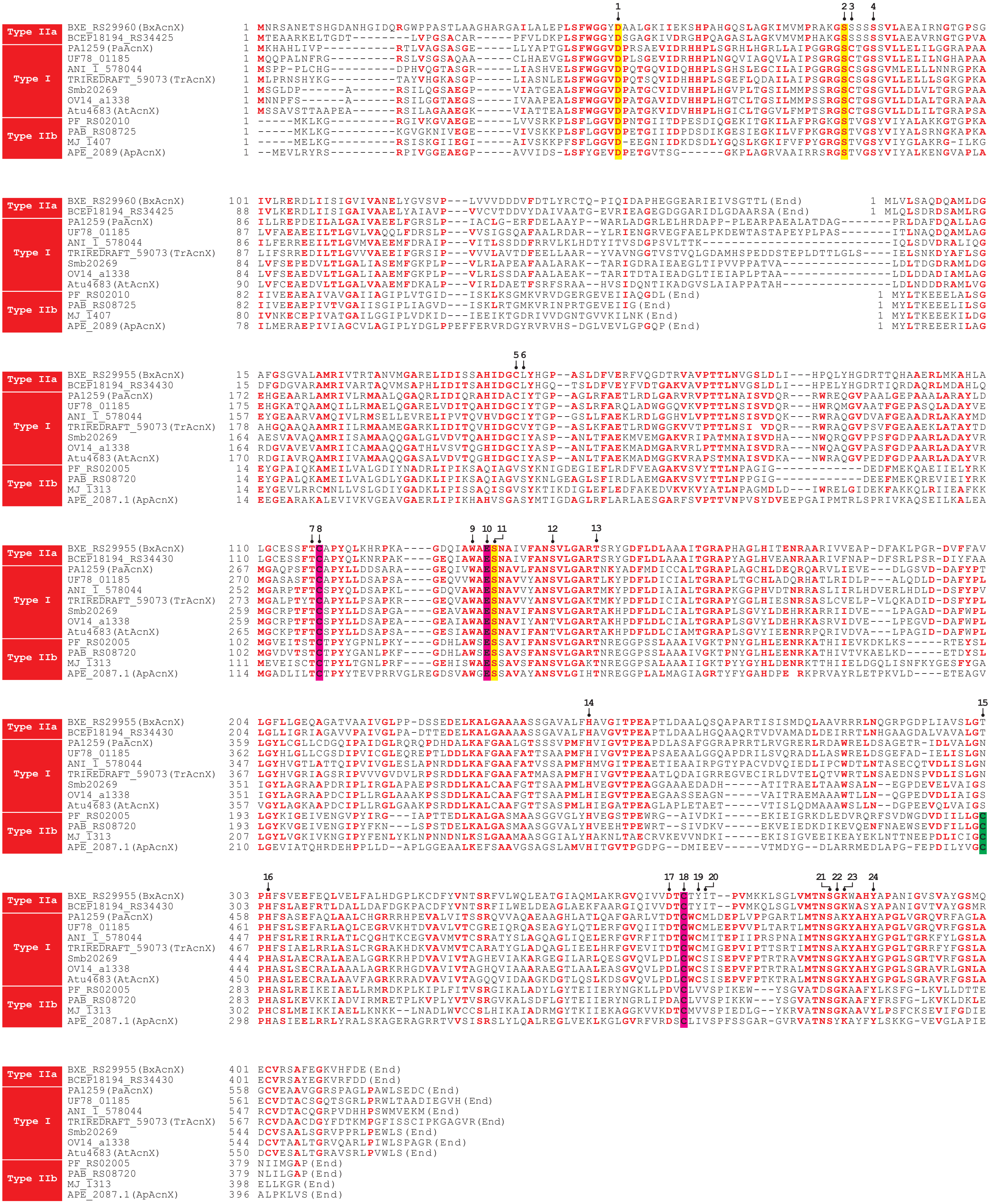

Figure S5. Multiple amino acid sequence alignments of AcnX. Red-bold letters indicate highly conserved amino acids.

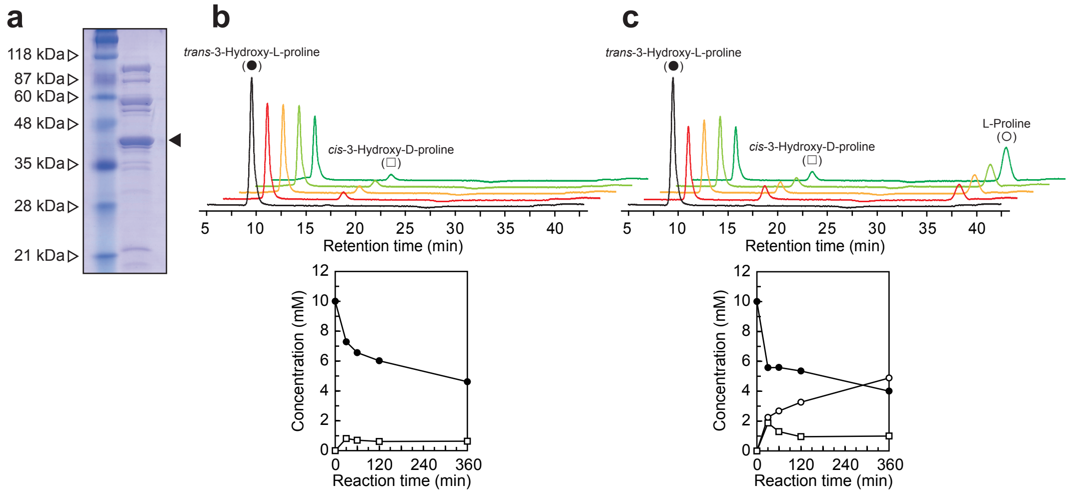

d

Specific activity (unit·mg protein^{-1})	K_m (mM)	k_{cat} (min^{-1})	k_{cat}/K_m (min^{-1}·mM^{-1})
0.229±0.014	23.2±0.05	28.7±0.2	1.23±0.01

Figure S6. Functional characterization of the PaLhpL protein. (a) SDS-PAGE analysis of recombinant protein (5 µg in a 12% (w/v) gel). Analysis of reaction products of trans-3-hydroxy-L-proline by PaLhpL (b) or PaLhpL + PaLhpH + NADPH (c) using an amino acid analyzer. Three independent experiments were performed, and the typical results obtained are shown. (d) Kinetic parameters as a trans-3-hydroxy-L-proline dehydratase. Values are the average ± SD, $n = 3$.
